# Social Disadvantages and Intergenerational Solidarity Views From Older Adults: A Qualitative Study

**DOI:** 10.1177/00469580221144621

**Published:** 2023-01-05

**Authors:** António Fragoso, Sandra T. Valadas, Liliana Paulos

**Affiliations:** 1 University of Algarve, Faro, Portugal

**Keywords:** older adults, family intergenerational solidarity, educational background, inequalities

## Abstract

In this article we aim at understanding the influence of social disadvantages on intergenerational solidarity. For this study, we have considered biographical research through narratives. These narratives help explain and reflect on the beliefs of the participants, implicit theories, and their life experiences. A snowball sampling technique was considered, and the data were collected by means of 58 narrative interviews with men aged between 60 and 93 years of age, living in urban and rural areas of southern Portugal. The interviews were conducted face-to-face in the participants’ houses or community centers. Content analysis was performed and our results indicated that a low educational background was determinant in the trajectories of these men. Unqualified or low qualified occupations and, in consequence, low financial capital, had both a direct and indirect effect on various domains of life. As a result, a significant part of the men live in poverty, and many others experience a very difficult situation. The results also revealed that social disadvantages had an impact on structural, functional, and associational solidarities. The levels of intergenerational exchanges are reduced and unsatisfying. Our study suggests that social class is a key factor in explaining the inequalities of older adults and also influences intergenerational solidarity at a family level.

**What do we already know about this topic?**
The accumulation of social disadvantages can lead individuals into a difficult situation that is beyond their control and harm various dimensions of their lives. Intergenerational solidarity can, potentially, counteract these negative influences through the exchange of resources between members of a family lineage.
**How does your research contribute to the field?**
Educational background is the primary source of perceived inequalities among the participants, which explains the levels of poverty that older men live in. Social disadvantages have a negative impact on intergenerational exchanges which are reduced and unsatisfying. Social class and social-economic difficulties seem to reduce solidarity within the family.
**What are your research’s implications toward theory, practice, or policy?**
New policies should be designed to deal with the poverty of older adults. It is advisable to redefine policies targeting the adult population and increase the qualifications of adults. These policies should be connected to existing practices, that is, measures to tackle these problems should be implemented not only during infancy and adolescence, but also during adult life.

## Introduction

The intense demographic aging of the world will soon have an impact on various dimensions of social life. The number of persons aged 80 years or above is projected to triple in 2050 and 1 in 6 people in the world will be over 65.^1^ The aging of the population will be especially intense in Europe. According to the European Union^2^ by 2050, half of the EU Member States are projected to have an old-age dependency ratio above 50.0% (less than 2 working-age people for every person aged 65 or older). The aging of the working population, the higher life expectancy, and younger generations entering later in the labor market, means that mainly 1 generation, that of medium age, is working in many European countries^3^: 2 of the 4 living generations in a family lineage are now retired, while the youngest is at school. In Portugal, the aging index has risen from 98.8, in 2000, to 182.7 in 2021, being one of the highest of the European Union.^4^ The projections show that in 2060, 34.6% of the Portuguese population will be aged 65 or older, with almost half of the older adults (46.5%) aged 80 years or more.^5^

In this scenario, older adults became a source of concern. Frequently described as a homogeneous group, but also as a problematic one,^6-8^ it is important to study the impact of individual, structural, and societal factors throughout the life course and, particularly, at an old age. Gender caught our attention because of its effect in participating in adult education and learning: on average, in the EU28, female participation is higher than male participation.^9^ Men are becoming the minority of participants in many learning spaces.^10,11^ Previous research^12,13^ indicated that promoting learning among older men, especially those with high levels of illiteracy, could make a difference in several scopes of their lives affecting, in a quite positive way, their well-being. These arguments were the basis of the European project focused on the learning of 60-year-old-men or older, which was used as a data source for this article.

This demographic scenario led supranational institutions and governments to embrace the concept of Intergenerational Solidarity (IS): briefly defined as social cohesion between generations^14-16^. It can be reflected at the societal level, but also at the group level and, more specifically, in families. At the societal level, political decision-makers and social scientists worry about the sustainability of the social security and pension system, as well as the increase in public expenditure via health systems. This has produced recurrent analysis^17^ of the financial and tax burden for younger generations, and is seen as largely excessive compared to that of previously older generations. With this reasoning, there is a serious imbalance in the flow of generational financing that can potentially cause conflicts between the generations. However, resources transferred between generations include much more than public economic exchanges. Care and various types of support, for example, are difficult to quantify and do not appear in this type of accounting, but can be analyzed within families.

Demographic changes seem to affect the structure of family networks, reducing the number of members and the distribution by age. The increase in life expectancy and the coexistence of multiple generations living over a broader timespan may reinforce intergenerational bonds, creating denser exchanges between family members.^18^ This means that solidarity between the members of a family lineage can potentially be positive and counteract some of the social disadvantages experienced by older men. Therefore, our aim is to analyze social disadvantage and understand its influence on intergenerational relations. Our research question can therefore be formulated in the following way: Can solidarity within the family have a positive effect on the lives of older men? We will focus mainly on the dimensions of the family level. Nevertheless, when relevant, we will reflect on the private-public relationships. We will begin by theorizing on social disadvantages and proceed to define micro-solidarities.

## Social Disadvantage

Generally speaking, social disadvantage refers to the difference that privileges or limits a certain social group, such as economic status, educational background, gender, and age, among others.^19^ Inequality reveals itself in access to basic rights but mainly in access to opportunities.

Primary factors causing social disadvantages are low levels of literacy^20^ and education. Occupation represents the major structural link between education and income. In turn, income relates directly to the material conditions that may influence health, regardless of employment status.^21^

The experience of simultaneous, permanent, or long-lasting disadvantages in more than 1 life domain further reduces people’s ability to manage everyday life.^22^ Also, the probability of experiencing simultaneous disadvantages in more than 1 life domain seems to be higher for the older age groups than for the younger age groups,^23-25^ which means that the accumulation of disadvantages could be amplified by old age vulnerabilities.^25^ This vulnerability of older adults to inequality has been gaining increasing attention.^9,23,26,27^ Several studies^21,28,29^ revealed that among older adults different indicators are associated with each other. For example, Avlund et al^30^ suggested that income and tenure which reflect material wealth were related to functional decline and death in both men and women.

Research on cumulative disadvantages has shown that financial capital in later life is determined by previous employment history and, therefore, influenced by educational level and occupational status.^31,32^ The existing evidence suggests that the amount of pension benefit depends on the individual employment history (eg, regular or atypical employment; frequency of employment interruptions), and the chance to save for retirement or to enroll in private pension savings depends on the level of income during working life.^33^ Therefore, earlier inequalities in educational attainment and in labor market position can lead to serious economic inequalities in old age.

More recently, Shaw et al^27^ demonstrated that several types of disadvantages are consistently associated with the probability of living alone including financial insecurity, never having been married, for women, and never having been married and mobility impairment, for men. Also, for older men, low education has become an increasingly strong determinant of living alone. These findings, from a repeated cross-sectional survey of Swedish adults aged 77+ during 1992 to 2014, suggest that older adults who live alone are a subgroup that is particularly, and in some cases increasingly vulnerable, with respect to social and functional status.

## Intergenerational Solidarity

IS seems to connect intrinsically both solidarity and conflict. Underlying solidarity is the notion that bonds are created between individuals and groups in such a way that affection, interaction, and providing assistance are intrinsic to this bonding. Solidarity and conflict are difficult to measure in social science research^15^ but solidarity can be at least understood qualitatively at the family level when we introduce questions to understand the relations between members of the family lineage, for example.

IS is understood within the context of shared expectations and obligations, which, at the micro-level, can be interpreted in the succession of generations within families. Six conceptual dimensions were used to understand IS^15,16^: (i) affectual solidarity—type and degree of positive sentiments held about family members, and the degree of reciprocity of these sentiments; (ii) associational solidarity—the type and frequency of contact between intergenerational family members; (iii) consensual solidarity—agreement in opinions, values, and orientations between generations; (iv) functional solidarity—the giving and receiving of support across generations, the exchanges of resources; (v) normative solidarity—expectations regarding filial obligations and parental obligations as well as norms about the importance of familistic values; and (vi) structural solidarity: the “opportunity structure” for cross-generational interaction reflecting geographic proximity between family members.

It was impossible for us to focus on all 6 components of IS. To characterize deeply some of these types of solidarity we would have to use quantitative instruments or scales. Therefore, we will focus only on those types of solidarity we have qualitative data on, namely associational solidarity, functional solidarity, and structural solidarity.

It is important to stress that structural solidarity affects functional solidarity, through geographical distance, and the mediating effect of some household characteristics (eg, health, socioeconomic status, and gender). Thus the “exchanges of support and distribution of resources may reinforce, not reduce, social inequalities as the most deprived and penurious in the poorest groups seem to receive less support.”^34^ This links strongly with social disadvantages, the social and economic status of people, and social class.^35^ Structural factors should not be overlooked.

Intergenerational relations are complex and can be extremely positive or extremely negative. The benefits of positive solidarity between generations appear frequently in the literature,^36^ but also customarily in publications focusing on aging as a chance for negative consequences or conflictive relationships between the generations.^37^ Again, it is difficult to separate solidarity from conflict. In a way, when solidarity fails or is absent, there is the possibility of conflict. The idea of solidarity/conflict implies that exchanges between generations can be imbalanced^38^ for different reasons. First, generational exchanges are not only determined by individuals but mediated by the state and the market.^39^ The distribution between who pays taxes, buys services, and gives or receives time or care is a function of many different factors, some of them established as state obligations of market rules. Over time economic and financial factors also have an influence on the amount of resources available to different generations. The imbalance of exchanges between generations can also be seen at the micro family level. Despite the fact that families are also influenced by macro factors (this is evident during a crisis), individual choices also matter. For example, grandparents often assume the care of grandchildren without knowing if their adult children would care for their parents in the future.^39^ Other studies demonstrate that the flow of resources like caregiving and social support, is higher downward than inward,^40^ although the intensity and regularity of these downward flows depend on the health and socioeconomic status of parents.^41^ It seems clear that IS comprises a number of interconnected dimensions. The lives of families cannot be separated from macro-factors such as the influence of the state or the market^39^; and there is a public and a private dimension of IS.^42,38^ Even if our focus, in this article, is on families, we should be able to produce some comments on the wider dimensions of IS.

## Methods

This is a qualitative study since our purpose is to understand people’s experiences from their own points of view.^43^ Biographical research is adequate to provide interpretations of subjects’ accounts regarding their past, present, and future^44^ and offer rich insights into the dynamic interplay of individuals and history, self, and other.^45^

We used narrative interviews to capture different aspects of the participant’s life course^46^: childhood (family background and schooling), working life, transition to retirement, and current situation (health status, financial situation, family, and social networks).

### Participants

We interviewed 82 men between 60 and 93 years old living in urban and rural areas of southern Portugal (region of Algarve).

The participants were arranged into 3 groups, based on educational level and context of life (rural or urban): (i) group 1 included men with low educational backgrounds from urban areas (n = 30); (ii) group 2 integrated men with low educational levels, but living in rural areas (n = 28); and (iii) group 3 was made up of men with higher educational background from urban and rural areas (n = 24). In this article, we only considered the results from group 1 and 2 (n = 58), due to our focus on social disadvantages.

More than half of the participants were married or cohabiting, the rest of them were widowed, divorced, and single. Most of them had between 1 and 5 children, and some of them had grandchildren, brothers/sisters, and nephews/nieces. Regarding educational background, many of the participants had 4 years of schooling or less (3 were illiterate) and a minority of them had between 5 and 9 years of schooling. Most of the participants were retired and during their active life they worked in the services and trade activities sector (eg, merchant and seller), artisans and qualified workers (eg, bakers and mechanics), and unskilled professionals (fishermen and construction workers). Overall, the participants had a lower socioeconomic status based on their education, income, and type of job.

### Instrument

As previously mentioned, we chose the narrative interview.^47^ Although it is an in-depth interview, a previous script was prepared to promote interaction with the interviewee and to obtain a greater wealth of data.^48^ The interview script was developed based on the literature review. The purpose was to understand how the older adults lived and prepared for retirement. It was developed in English by one of the teams that participated in the project (Anonymous) and then it was translated into Portuguese and adapted to the Portuguese reality. In a second phase, the script was analyzed by 2 experts, considering “clarity,” “adequacy,” and “relevance.” It was tested with 4 older men. Some changes were made, namely, the modification of concepts that were not adequate and the reformulation of items with a dubious or unclear meaning. The final version included 4 themes: (i) the professional pathway and experiences; (ii) the preparation for retirement; (iii) the dynamics and relationships with family and friends, their neighborhood, and socialization; and (iv) the participation in the community, problems identified, and suggestions.

### Procedures

Regarding ethical issues, all the participants were voluntary, and none of them received any kind of incentive to participate in the study. The participants signed an informed and free consent statement where they were informed about all the study-related issues (eg, aims, methods, sources of funding, and institutional framework), anonymity, confidentiality, researcher’s duties, and responsibilities. The acronym PART (1, 2, 3. . .) means participant and was used to guarantee anonymity when we did match the quotes to the participant.

A snowball sampling technique was followed. The first contact with the participants was made in person through the support of civil society organizations (eg, day centers and community centers). From the network of contacts of these older men, we obtained other participants. These new participants were contacted by telephone and all of them accepted to participate in the study. The selection considered age and gender (men aged 60 years or more) and respecting the heterogeneous nature of the elderly (work status, education, income, marital status, and their participation in civil society organizations).

The empirical saturation technique was applied.^49^ The inclusion of new participants was suspended based on the data that had been collected or analyzed hitherto, further data collection and/or analysis were unnecessary. Data collection was carried out between May and December of 2018 and the interviews were conducted face-to-face. Most of the interviews took place in the participants’ residences. However, some were conducted in private spaces in the institutions of civil society through which the participants were contacted. Each interview lasted between 90 and 150 minutes. In some cases, we conducted 2 interviews per participant. All interviews were audio-recorded, previously authorized by the participants, so that the transcription of the information was as reliable as possible.

We used qualitative content analysis^50^ to understand meaning^51^ because it allows us to define categories through an inductive procedure. We searched for emergent patterns of meaning^52^ that could guide us in building categories. First, we transcribed the interviews and returned the transcripts to each participant (only to literate participants who shared an email address) to validate the information. After the transcription, collection, and building of a corpus of analysis (resulting data), we carried out a floating reading of the interviews^53^ to find commonalities between the responses. We then created a coding frame for each group, inductively, in a data-driven way. This means that the interview transcripts were read to understand how the older adults' social disadvantages influenced intergenerational solidarity.

## Findings

### Social Disadvantages

Several conditions limit both groups of men who were interviewed in the research, but these are primarily related to their educational background. A significant number of older men have low levels of literacy. Consequently, most of them had unqualified or manual jobs for most of their lives. These are the type of insecure jobs that brought them a very low financial capital and impeded them from saving up money over the years. Due to financial shortage, some men are today in their old age dependent on assistance and solidarity to get their meals and have at least a room to live, as illustrated by this quote: “*I live with great economic difficulties. . . (pause) after a lifetime of work that guaranteed me a comfortable life. Today, I got food from the (Community) Centre because the disability pension was refused*” (PART19, 64 years old, single, 4th grade, rural area).

As some have worked informally for long periods of their lives (pensions systems based on working contributions were only created after democracy) they receive pensions around or below 200€, in a country where the minimum wage is about 665€ and renting a very small flat in a peripheral area costs at least 300€/month. Hence, some need to work even after retirement for survival purposes. The intensity of financial difficulties is higher in urban areas than in rural areas.

So, previous inequalities in educational achievement and position in the labor market can lead to severe financial inequalities in old age. The men’s low financial capital had a direct influence on at least 2 other life dimensions: health and social networks. Although the national health system is mainly free, some medical areas of expertise are absent from health centers and hospitals or imply long waiting periods. Critical or sudden health problems have further consequences difficult to deal with and can reduce men’s mobility and compromise their ability to socialize, therefore contributing to the shrinkage of social networks. Some still have families, but a significant number are living alone and do not meet family members regularly. Mobilizing various types of resources is quite difficult for these men, and loneliness is a severe problem that affects many of the men interviewed:

My son and daughter-in-law are my biggest support. I have no relationship with other family members who live close by. They only visited me when I was hospitalized. I don't have neighborhood relations because I do not have neighbors anymore. In the past, people lived together more, neighbors met on the street. I often feel lonely, when I feel like this, I watch TV until I fall asleep (PART17, 84 years old, widower, illiterate, urban area).

Educational background was determinant in these men’s trajectories, leading to unqualified or low-qualified occupations. Consequently, low financial and social capital seems to be a “death sentence” difficult to surpass, along with amplified old-age vulnerabilities. Experiencing disadvantages simultaneously, and in more than 1 domain, diminishes the capacity to have a dignifying life, leading to the accumulation of disadvantages, very complicated to disentangle.

### Intergenerational Solidarities

Group 1 includes older men from urban areas with low educational backgrounds and economic status, living in very precarious conditions and frequently suffering from health problems. Our interviews allowed us to analyze the effects of these conditions on the various types of intergenerational solidarities.

Regarding structural solidarity, among these men, the interaction with their families is very poor and unsatisfying. Because of their low income, we find a minority with a driver’s license and none has a car. This can represent a serious constraint in terms of mobility when public transportation is working deficiently. Their interactions with adult children or grandchildren are scarce. New communication technologies could eventually facilitate contacts or help with some kind of support. But because of their economic status, none of the men we interviewed use computers or other digital devices. Also, not all possess a mobile phone, and the ones who do, make a basic use of it. This means that geographical distance constrains today’s cross-generational interactions.

When we analyze social contacts and shared activities between intergenerational family members (associational solidarity), loneliness is a serious problem that affects most of the men in this group. Many of them are widowers and living alone, in some cases in a small precarious “room” rented by a friend. There are others that do not have a place to stay at all. Some of them have families, but they do not meet family members frequently.

I have a daughter, two grandchildren and a brother. My daughter works nearby, and I'll meet her at work if she doesn’t come to see me. I don’t usually hang out with my grandchildren. My youngest grandson is two years old, and I do not know him yet. When I was sick, my ex-wife and brother visited me, but my daughter never came to see me. My sister-in-law is a person who cares about me, who asks me how I am doing, if everything is okay. (PART 28, 62 years old, divorced, 5th grade, urban area)

Generally speaking, many of these men have health and mobility problems, being confined to the area of their neighborhood. This means that they are destined to wait for the visits of their adult children and grandchildren or are limited to phone calls. The major part of their socialization is not intergenerational, but among people of similar age groups and use of the public spaces of the cities. Contacts with neighbors are “civilized” but not deep, and friends are scarce:

I have a young neighbor who helps me with what I cannot do (shopping, going to the pharmacy). I do not have relationships with the neighborhood, because it is all new people. In the past, people would gather at the door and talk, get involved in the community. I would like to make new friends, before I participated in activities (going to the theater), but as I have mobility difficulties I stopped participating. I'm used to being alone, I watch TV, I call friends, old co-workers (PART 21, 68 years old, single, 6^th^ grade, urban area).

Functional solidarity is characterized by intergenerational exchanges of support and resources (eg, financial, physical, and emotional) but, once again, the primary conditions of these men seem to be the basis of a low level of exchanges. During their working life, the scarcity of their financial resources meant that there was not much to share with their children or adolescents, nor, later, with their adult children or grandchildren. It could eventually happen that inward economic exchanges would be predominant at present. But our interviews show that only a minority of these older men have financial support from the younger generations. The same happens with care: only a few receive it from their adult children whereas a significant group goes to day-care centers to get their meals, hygiene, help with medication, and company from other older adults. So, functional solidarity is characterized by a low level of intergenerational exchanges of all sorts. We have not identified in our interviews signs of overt conflict between generations, but often these men show signs of sadness, bitterness, and even depression.

Group 2 includes older men from rural areas with low educational backgrounds and economic status. Their financial difficulties are of lower intensity when compared to older urban men. The context functions as a buffer that impedes falling under the threshold of poverty. A significant part has occupations related to subsistence agriculture: either they have small rural properties that provide basic food and some informal complementary income, or they have manual occupations related to the rural world—also providing some complementary income.

Social and geographical space is crucial to analyze associational and structural solidarity. These men and their families live in isolated communities or small fragmented clusters of houses spread throughout the country, in a mountainous area with deficient infrastructures, lack of services of all types (educational and health services being the most salient) and public transport is almost nonexistent.

The “mountainous identity” includes a fatalist dimension that internalizes social and economic decay as a collective fate—there is no future in those areas. Quite often families assume it to be a major task of life to encourage their children to get an education, look for a job somewhere else and never come back. This means that structural solidarity is closely connected with associational solidarity: geographic lack of opportunity is the major factor that leads to the low frequency of contact between parents and their children, which moreover reduces as children grow older and migrate. Also, the lack of opportunities for leisure in mountainous communities is such that shared activities are in most cases reduced to a minimum. Almost all adult children studied (some, but not much, in higher education) and later found jobs very far away from their parents. In these situations, contacts are limited to some weekends from time to time. Soon, this new generation “visit” their parents and later on “visit” their grandparents when possible.

Therefore, functional solidarity is based on uneven exchanges that become eroded as time goes by. There was a considerable flow of downward exchanges, which were financial, care, and support, from parents to children and adolescents, but the young adult’s migration searching for jobs in urban areas impedes the reciprocity of such flows. While it is more common that the 2 older generations continue to live in mountainous communities, the younger ones represent those who migrated. The opportunities that this younger generation has to make concrete inward exchanges are indeed very limited. As happened with group 1, we have not identified an overt conflict between generations, or older men complaining about the support and care they get from younger generations. Quite the opposite: although older men do not like their solitude, they assume there is no way out and that younger generations should go on with their lives.

In short, the situation of older adults in these mountainous communities is characterized by small social networks and low social capital, isolation, loneliness, and lack of opportunities even to socialize within their own age groups. Access to health services, mobility, and access to care are serious problems that even public policy cannot handle. State or private care services for older adults are distant.

## Discussion and Conclusion

Our results revealed that illiteracy and educational background are the primary sources of inequalities, as previously shown by Machin.^20^ This has been a very common and well-known fact for decades. However, less common is to trace the direct and indirect consequences of primary factors such as education and financial capital over the life course to explain how complex is the situation of older adults who were exposed to cumulative disadvantages. We have shown that occupation is a major link that determines income which, in turn, has a great deal of influence on the older adult’s health,^21^ the incapacity to save for retirement^33^ and, as supported by other studies,^27^ in increased vulnerability to living alone. The theory of cumulative disadvantages has therefore a high degree of utility, particularly in explaining how serious the situation of older men is.

On the other hand, it is striking that public social security is unable to deal with people’s extreme frailty and poverty, questioning the existing flows between private and public life. An important part of the resources exchanged between generations, either inward or downward, stays in the private domains of life and functions as a positive support when complemented by the resources exchanged via public domains. But the men we interviewed belong to the working class. Their financial capital does not allow a sufficient distribution of private resources between generations throughout their life course. As a consequence, at an old age, the private inward exchanges are also not sufficient to assure them a dignified life and thus they depend on the state. If historically family solidarity was substituted by public solidarity, it seems that in Portugal the state provision in a neoliberal context has made this promise in vain. We therefore agree with Bawin-Legros and Stassen: 42 it seems that the public transfers which assure the relative autonomy between adult generations are weakening and the private sector is growingly affirming its importance.

This means that, in the context of IS, relations between the state and the citizens are not uniform or universal. Our results show that the public systems are unevenly used by people according to social class. A minimal state that reduces its provision to citizens affects many more people from the working class—middle classes have financial resources to turn to the private sector, when needed. Regarding intergenerational solidarity, this effect was previously pointed out by Künemund.^54^

The analysis of the interviews makes clear that social class is central in explaining the situation of older adult men. It further suggests that intergenerational solidarity at the family level is largely influenced by social class. Although not much of the literature talks about social class, gender, or ethnicity regarding older adults, the study made by Timonen et al,^55^ in Ireland, concluded that intergenerational solidarity, at the family level, is strongly contoured by socioeconomic status and that intergenerational solidarity within families is also shaped by the welfare state.

As a first conclusion, we should stress that the perceptions of older men in our sample allowed us to understand the devastating role of social disadvantages in the aging process. More, it explains the persistence of poverty in working-class groups at an older age, especially serious when combined with the inefficacy of the welfare system. Our second conclusion relates to IS. Our results indicate that people in poverty have unsatisfying intergenerational solidarity levels. We would expect that IS solidarity could potentially compensate for the difficult situation these men live in. But actually, we have revealed the opposite. This also seems to give strength to researchers that claim that intergenerational solidarity may even mask the causes of inequalities. For example, Timonen et al^35^ argue, as Kohli,^56^ that highlighting the generational conflict as the new basic cleavage in society tends to contain other inequalities and may distract attention from still-existing problems of poverty and exclusion.

Our conclusions have implications for policy and practice. In order to prevent the destructive effects of social disadvantages on working-class older adult groups, new policies should be designed to deal with the poverty of older adults. As low educational background is the basic factor that determines these inequalities, it is advisable to redefine policies targeting the adult population and increase the qualifications of adults. These policies should be connected to practice because measures to tackle these problems should be implemented, not just during infancy and adolescence, but also during adult life. Instruments to assess poverty risk during adult life should be created in order to allow coordinated action between the state and civil society organizations.

We have to consider the limitations of the investigation. As previously mentioned, the original purpose of the interviews was to understand how older adults live and prepare for retirement. In consequence, the interviews were not fully adequate for the central purpose described in this paper, and it is possible that we miss some information. A second limitation connects with the impossibility to consider in this paper the group of highly educated men (we do not have the necessary space), which would show the other side of the social reality we described—like a mirror. A third limitation refers to the fact that we have only interviewed men. We have not collected women’s perceptions, nor the perceptions of some other members of the family lineage. This advises that in the future we can design more complete research that can shed more light on this complex issue.
